# Reviewed article: Santos SGP, Cruz CFR, Prezotto KH, Moreira RC, Galdino MJQ, Oliveira RR, Scholze AR, Melo EC. Temporal trend and spatial analysis of breast cancer mortality in Paraná, 2012-2021. Epidemol Serv Saude. 2025:34;e20240171

**DOI:** 10.1590/S2237-96222025v34e20240171.a

**Published:** 2025-09-08

**Authors:** Cristine Vieira do Bonfim

**Affiliations:** 1 Fundação Joaquim Nabuco, Recife, PE, Brazil

Peer review A

## Questionnaire

Does the manuscript contain new and significant information to justify publication? No

Does the Abstract (Summary) clearly and accurately describe the content of the article? No

Is the problem significant and concisely stated? No

Are the methods described comprehensively? No

Are the interpretations and conclusions justified by the results? Yes

Is adequate reference made to other work in the field? Yes

## Manuscript Structure

Length of article is: Too long

Number of tables is: Adequate

Number of figures is: Adequate

Please state any conflict(s) of interest that you have in relation to the review of this paper. None

Interest: Good

Quality: Average

Originality: Average

Overall: Good

Recommendation: Major revision

## Comments

Agradeço o convite para realizar a revisão do manuscrito. Aproveito para parabenizar os(as) autores(as) pelo trabalho. Trata-se de um estudo ecológico que teve por objetivo analisar as características sociodemográficas, a tendência temporal e a distribuição espacial da mortalidade por câncer de mama no Paraná. Observou-se tendência de crescimento das taxas de mortalidade e presença de hotsposts em macrorregionais do estado. Há coerência entre métodos e resultados. Pondera-se que os resultados são relevantes, porém o estudo necessita ainda de alguns ajustes, conforme descrito abaixo.

Comentários maiores

No título sugere-se a inclusão do desenho do estudo e do período de referência, uma vez que a tendência temporal é um elemento essencial no trabalho. Outra sugestão consiste em retirar o termo estado.

Resumo

No desenho ecológico é essencial informar a unidade de análise.

Incluir a fonte de dados e os indicadores utilizados, uma vez que a análise foi realizada com as taxas e não com o número.

Sugere-se que as técnicas de análise temporal e da espacial, sejam descritas em frases separadas e de forma mais compreensível para o(a) leitor(a) da RESS.

Nos resultados a primeira informação deve consistir no número de observações do estudo. Nota-se a ausência dos dados numéricos. Trata-se de um estudo epidemiológico, logo os dados são necessários na seção de resultados. O texto atual está muito semelhante ao de uma discussão.

Em relação ao resumo é que a seção de métodos não possibilita a compreensão dos resultados, essas duas seções precisam ser mais harmônicas.

A conclusão considerou a tendência, porém precisa envolver também a análise espacial.

Em relação aos descritores, sugere-se que sejam mais relacionados ao estudo. Enfermagem e monitoramento epidemiológico não se relacionam diretamente ao tema estudado. Talvez os seguintes descritores retratem melhor o estudo. Por favor, avaliem: Análise Espacial; distribuição temporal ou série temporal; e estudos ecológicos.

Introdução

Primeiro parágrafo, os dados precisam ser comparáveis. Então sugere-se que sejam apresentados o número de casos e a taxa, igualmente para a mortalidade. Apresentar o número de óbito e a taxa de mortalidade.

Segundo parágrafo necessita da inclusão de dados epidemiológicos, que demonstrem aumento. A segunda frase, estaria mais adequada no primeiro parágrafo.

Terceiro parágrafo em nenhum dos anteriores, foi informado que o câncer de mama, ocupava a primeira posição no ranking mundial.

Nota-se a ausência na seção de trabalhos sobre tendência temporal e a análise espacial envolvendo o câncer de mama. Importante incluir estudos que evidenciem as contribuições dessas análises.

Importante incluir a justificativa antes do objetivo, que demonstre a relevância, a contribuição do estudo e a sua inovação (se houver).

No objetivo cita-se características sociodemográficas, porém o SIM, tem no máximo a variável escolaridade que pode ser considerada social. As demais são demográficas. Será que demográficas ou até mesmo epidemiológicas, não ficaria mais adequado? Sugere-se retirar o termo estado e incluir o período de referência.

Métodos

No desenho do estudo a unidade de análise precisa ser revista. Inicia-se informando que se trata de um estudo de tendência temporal, porém a unidade de análise se refere ao espaço (município). Considerando que é um estudo de análise temporal, a unidade deve ser uma medida de tempo. Importante justificar a escolha dessa unidade e incluir as limitações dela na seção de discussão.

Por favor, avaliem se este não é um estudo ecológico misto.

A população do estudo necessita ser descrita detalhadamente. Embora, seja raro o câncer de mama também pode acometer indivíduos do sexo masculino. Esses indivíduos foram incluídos no estudo?

Terceiro parágrafo, o estudo foi desenvolvido com dados secundários. Logo, não houve coleta de dados. Para estudos com dados secundário, deve-se utilizar o termo fonte de dados.

O processo de obtenção dos dados, precisa ser descrito detalhadamente. Incluir a data de acesso aos dados e colocar o link completo na lista de referências.

O período de referência do estudo precisa ser mais bem descrito. Qual o motivo para escolher o ano de 2012? O que houve de relevante? Sobre o período final do estudo, é necessário considerar a existência dos anos de 2020 e 2021, que foram afetados pela pandemia pela COVID-19. Todos os indicadores de saúde foram afetados, isso pode ocasionar algum viés na análise temporal.

Em estudos com dados secundários a completude é essencial. Importante incluir a informação sobre a completude das variáveis analisadas.

Sobre a variável raça/cor da pele o IBGE utiliza pretos e não pretos (pardos + negros), por que foi utilizado uma agregação diferente?

A análise temporal pode ser mais bem descrita, inclusive com referências. Precisa ser descrita para o(a) leitor(a) da RESS que não especialistas, consigam compreender.

As malhas cartográficas do IBGE devem ser descritas junto com as demais fontes de dados.

O processo de obtenção dos dados, pode ser descrito, com mais detalhes para possibilitar o seu entendimento. Houve casos que não foram ser georreferenciados? Se sim, quantos?

A forma de cálculo dos indicadores precisa ser incluída na seção.

Resultados

Os resultados devem ser apenas descritos. Deve-se evitar interpretações. Por exemplo: houve predominância.

A seção de resultados está demasiadamente longa. Pode ser reduzida, sem prejuízo, somente com destaque para as principais informações no texto.

A descrição dos resultados com o nome dos municípios, deixa um estudo com um caráter muito local. Os(as) leitores(as) da RESS não conhecem e essa seção está bem detalhada, nesse aspecto.

Na página 8, primeiro parágrafo são apresentados dados que não constam nas tabelas ou figuras. Precisam ser incluídos ou removidos do texto.

Na descrição das figuras. Nota-se a ausência da descrição da figura 1C. Além disso, cada figura deve ser descrita de forma separada.

Para a Figura 2, somente a letra C está descrita no texto. Todos os resultados precisam ser descritos.

O título da Figura 2, menciona taxa de incidência, porém esse não é objetivo desse estudo. Sugere-se que os resultados se concentrem na mortalidade.

Discussão

A seção está demasiadamente longa, pode ser reduzida sem danos ao seu conteúdo.

O primeiro parágrafo deve iniciar com uma síntese, sem dados numéricos, dos principais resultados.

Observa-se a presença de vários parágrafos que não fazem conexão com o resultado. O texto fica bastante semelhante ao de uma revisão. A discussão consiste no relacionamento dos resultados com a literatura.

A discussão sobre a raça/cor da pele, está bem confusa. Poderia ser mais compreensível, refletindo somente sobre o resultado do estudo.

Trata-se de um estudo de tendência temporal, esse deveria ser o foco da discussão, porém boa parte é dedicada a discutir a menor ou a maior taxa.

Nas limitações do estudo, é importante incluir a unidade de análise.

Sugere-se separar as limitações das conclusões do estudo.

Referências

Observa-se um excesso de referências institucionais. Algumas podem ser substituídas por artigos científicos.

Tabelas e figuras

Tabela 1 - sugere-se iniciar com a parte descritiva, número de óbitos e taxa de mortalidade, depois APC, IC e p-valor e finalizar com a tendência.

Para o título - Distribuição das características demográficas dos óbitos, tendência temporal das taxas de mortalidade (por 100 mil) por câncer de mama em mulheres, Paraná, 2012 a 2021

Incluir as siglas e as abreviaturas abaixo da tabela.

Tabela 2 - taxa de moralidade por câncer de mama em mulheres (por 100 mil) segundo macrorregionais, Paraná, 2012 a 2021.

Figura 1 - Série temporal das taxas de mortalidade por câncer de mama (por 100 mil) em mulheres, macrorregiões de saúde, Paraná, 2012 a 2021.

A RESS publica em escala de cinza. Ajustar as figuras.

Figura 2 - o título da figura deve considerar as letras.

Comentários menores

Introdução

Primeiro parágrafo da introdução, a primeira frase necessita dos dados epidemiológicos, para que evidente o que está sendo denominado de elevadas taxas. Pondera-se que não existe uma estratificação para que as taxas sejam classificadas em alta, média ou baixa.

Segundo parágrafo, especificar quais são as estatísticas que estão se referindo.

Sugere-se para a descrição de todas as taxas, que se utilize apenas uma casa decimal após a vírgula. Também seria interessante padronizar a forma de apresentação. Substituir a (/) por (por).

O terceiro parágrafo é constituído por uma única frase. Igualmente o quarto parágrafos. São frases extensas, com muitas informações, que tornam a leitura difícil. Sugere-se uma redação com frases mais diretas, utilizando mais os pontos do que as vírgulas, que agregam muitas ideias.

Quarto parágrafo, sugere-se iniciar o texto diretamente, com a retirada do “com enfoque”.

Métodos

Sistema de Informações sobre Mortalidade.

Na página 5, o quarto, quinto e sexto parágrafos, são constituídos por uma única frase.

Ao longo de todo o texto, é observada a presença de parágrafos de frase única. Solicita-se que sejam revisados, para uma melhor compreensão do texto.

Substituir raça/cor por raça/cor da pele.

Resultados

Sugere-se que a redação seja realizada, com o tempo verbal no passado.

Página 8, primeiro parágrafo, substituir o temo “categorias” por “variáveis”.

Discussão

Primeira linha, retirar o termo encontrados.

A redação deve ser realizada no impessoal.

O estudo necessita de uma revisão linguística

